# The disease burden of human cystic echinococcosis based on HDRs from 2001 to 2014 in Italy

**DOI:** 10.1371/journal.pntd.0005771

**Published:** 2017-07-26

**Authors:** Toni Piseddu, Diego Brundu, Giovanni Stegel, Federica Loi, Sandro Rolesu, Gabriella Masu, Salvatore Ledda, Giovanna Masala

**Affiliations:** 1 National Reference Laboratory of Cystic Echinococcosis, Istituto zooprofilattico sperimentale della Sardegna, Sassari, Italy; 2 Territorial Department of Nuoro, Istituto zooprofilattico sperimentale della Sardegna, Sassari, Italy; 3 Department of Political Science, Communication Sciences and Information Engineering, University of Sassari, Sassari, Italy; 4 Epidemiological Veterinary Regional Observatory, Istituto zooprofilattico sperimentale della Sardegna, Sassari, Italy; Texas A&M University College Station, UNITED STATES

## Abstract

**Background:**

Cystic echinococcosis (CE) is an important neglected zoonotic parasitic infection belonging to the subgroup of seven Neglected Zoonotic Disease (NZDs) included in the World Health Organization’s official list of 18 Neglected Tropical Diseases (NTDs). CE causes serious global human health concerns and leads to significant economic losses arising from the costs of medical treatment, morbidity, life impairments and fatality rates in human cases. Moreover, CE is endemic in several Italian Regions. The aim of this study is to perform a detailed analysis of the economic burden of hospitalization and treatment costs and to estimate the Disability Adjusted Life Years (DALYs) of CE in Italy.

**Methods and findings:**

In the period from 2001 to 2014, the direct costs of 21,050 Hospital Discharge Records (HDRs) belonging to 12,619 patients with at least one CE-related diagnosis codes were analyzed in order to quantify the economic burden of CE. CE cases average per annum are 901 (min—max = 480–1,583). Direct costs include expenses for hospitalizations, medical and surgical treatment incurred by public and private hospitals and were computed on an individual basis according to Italian Health Ministry legislation. Moreover, we estimated the DALYs for each patient. The Italian financial burden of CE is around € 53 million; the national average economic burden per annum is around € 4 million; the DALYs of the population from 2001 to 2014 are 223.35 annually and 5.26 DALYs per 10^5^ inhabitants.

**Conclusion:**

In Italy, human CE is responsible for significant economic losses in the public health sector. In humans, costs associated with CE have been shown to have a great impact on affected individuals, their families and the community as a whole. This study could be used as a tool to prioritize and make decisions with regard to a surveillance system for this largely preventable yet neglected disease. It demonstrates the need of implementing a CE control program aimed at preventing the considerable economic and social losses it causes in high incidence areas.

## Author summary

CE is a neglected tropical disease that remains a considerable health problem in endemic regions and which leads to substantial economic losses for agriculture sectors and public health systems. The parasitic cycle requires a definitive host (canidae) and an intermediate host (livestock species), while humans are only occasionally hosts. CE is more prevalent in areas where extensive or semi-extensive farming of livestock (mostly sheep) is common. The study of prevalence and health expenditure at the national macro-regional and regional level is a prerequisite for creating awareness of the importance of this disease. The Italian Ministry of Health provides remuneration to its regions’ hospitals for treatment of illnesses. In this study 21,050 hospital discharge records with CE diagnosis from 2001 to 2014 related to 12,619 patients were analyzed. The median of CE hospitalizations per annum was 848. The direct costs of HDRs for echinococcosis were estimated to be € 53,175,934.34. The assessment of economic remuneration by Italian Regions grouped by Nomenclature of Territorial Units 1 (NUTS1) shows that Southern Italy and its major islands spent €13,523,999 and €15,171,739 respectively. The national annual average direct cost for 10^5^ inhabitants was € 6,398. The study of the National DALYs, a measure of social burden of disease, shows that 41% was correlated to Island patients DALYs per annum are 223.4. An analysis of direct costs and an estimate of DALYs are key factors for establishing how to prioritize control measures for CE, which is essentially a preventable disease.

## Introduction

Cystic echinococcosis (CE), caused by the larval stage of the taeniid tapeworm *Echinococcus granulosus* sensu lato, is an important zoonotic disease with a worldwide distribution. The World Health Organisation (WHO) included CE on the official list of the 18 Negleted Tropical Diseases. In addition to this group, the WHO has identified CE as belonging to a subgroup of seven endemic or ‘‘neglected zoonotic diseases” (NZDs); these diseases are common where poverty, reliance on livestock or wildlife for social and financial capital, poor resilience, and the close proximity of people and their animals favor transmission[1] [2] [3]. Human CE remains highly endemic in pastoral communities, particularly in regions of South America, the Mediterranean littoral, Eastern Europe, the Near and Middle East, East Africa, Central Asia, China and Russia [4] [5] [6]. Domestic transmission of the infection relies on dogs as definitive hosts and a range of livestock ungulate intermediate hosts, mainly sheep and cattle [7] [8]. In the human host, the accidental oral ingestion of *E*. *granulosus* eggs may cause the development of cysts in many anatomic sites [9] [10].The initial phase of the primary infection is always asymptomatic. Small, well encapsulated, non-progressive or calcified cysts typically do not induce major pathology, and patients may remain asymptomatic for years or permanently [11]. The induction of morbidity depends on the number, size, and developmental status of the cysts (active or inactive), the involved organ, the localization of the cysts within the organ, the pressure of the cysts on the surrounding tissues and structures, and the defense mechanisms of the infected individual. Clinical signs may occur after a highly variable incubation period of several months or years [7] [12].

The disease represents a serious human and animal health concern that leads to significant economic losses derived from the costs of medical treatment, morbidity, life impairment and fatalities in human cases [13] [14]. CE is widespread in many regions of the world, affecting over 1 million people worldwide [15] [16]. Present estimates indicate that its global burden is about 184,000 DALYs (0.98 DALYs per case) [17]. The global financial burden of this disease in estimates of purchasing power parity is 4.1 billion international dollars annually, of which 46% is due to human treatment and morbidity and 54% is associated with animal-health costs [18] [19].

The reports from the EFSA and the ECDC highlight that a surveillance system on human CE is absent in Italy. As a result, no official data are transmitted to European authorities [20]. Due to the lack of official data, the Italian National Reference Centre for Echinococcosis (CeNRE) analyzed Hospital Discharge Records (HDRs) drawn from the National Ministry of Health. A retrospective epidemiological study of HDRs for CE cases carried out between 2001 and 2012 in Italy indicated that in Italy human CE continues to be a significant public health problem, particularly in the Islands and in the South. The study revealed an increased risk of human CE in correlation with “rural areas with comprehensive development problems” and within areas where sheep breeding is widely practiced [21]. The aim of our study is to perform an analysis of human echinococcosis disease costs in Italy; in particular, to calculate the economic burden of hospitalization costs and treatment and to estimate the Disability Adjusted Life Years on cases from 2001 to 2014. Objective estimates of the burden of CE are important because its underestimation downgrades its relevance to policy-makers and funding agencies [22] [23].To our knowledge, this study was the first of its kind to be performed in Italy.

## Materials and methods

### Ethics statement

In compliance with the current Italian legislation on privacy, it should be noted that the publication and or dissemination of HDR’s data and their processing must take place only in aggregate form.

### Study area

Italy is a country in South-Central Europe, occupying a peninsula and two big islands (Sicily and Sardinia) in the center of the Mediterranean Sea, with around 60 million inhabitants. Moreover, it is the 5^th^ country by population on the EU list (comprised of 28 countries) and 8^th^ on the 2015 World Bank ranking list of countries by their gross domestic product (GDP) [24]. In order to subdivide its territory, we adopted the Nomenclature of Territorial Units for Statistics (NUTS) 2010 classification (Eurostat, Regulation EC 1059/2003; European Parliament and of the Council, 2003b): a geocode standard for referencing the administrative divisions of countries for statistical purposes [25]. Italy is divided into 20 administrative units: the regions (NUTS 2). Regions are further grouped into five “macro regions” (NUTS 1). The Italian NUTS 1 are the North West (Valle d’Aosta, Liguria, Lombardia, Piemonte) with 16,131,000 inhabitants, the North East (Emilia-Romagna, Friuli-Venezia Giulia, Trentino-Alto Adige/Südtirol, Veneto) with 11,654,000 inhabitants, the Center (Lazio, Marche, Toscana, Umbria) with 12,071,000 inhabitants, the South (Abruzzo, Puglia, Basilicata, Calabria, Campania, Molise) with 14,168,000 and the Islands with 6,759,000 inhabitant (Sardegna and Sicilia) [26].

### Hospital discharge records

The data used to perform the study on the economic and health burden of CE were obtained from HDRs. The HDRs data regarding the period from 2001 to 2014, in anonymous form and free of personal information, were provided to CeNRE by the Italian Department of Health. In Italy, Since January 1^st^, 2009, the 2007 version of the International Classification of Diseases, Ninth Revision, Clinical Modification (ICD-9-CM) for encoding diagnosis and procedures contained in the HDRs has been adopted as the standard.

The HDRs summarize the medical records and classify diseases according to the ICD-9-CM and are divided by Disease Related Group (DRG), Major Disease Classification (MDC), type (medical or surgical) and description of DRG [27]. The HDRs contain an anonymous individual code for tracking patient’s hospital admissions, discharges and readmissions. Each anonymous individual code identified one patient. Specifically, our database include: patient’s admission and discharge dates, gender, age, domicile code, primary and secondary diagnoses codes, type of treatment (surgical or medical), length of stay in the hospital and mortality data. Data about hospitals include: regional and province code, department code, ward, type of hospitalization including ordinary hospitalization (OH) or day hospitalization (DH). OH refers to procedures that require an overnight hospital stay, while DH refers to procedures that require in-hospital treatment without an overnight stay. S1 Table describes all fields included in HDR schema.

The HDRs were converted to MySQL relational database management system (RDBMS) tables; calculations of cost elements were been performed as queries. The data, received as text file, were recorded in Microsoft Office Excel spreadsheets for better readability and in order to provide a graphic display of data and of grand total calculations. After integration of all information into the database, an extensive data checking and verification of completeness and compliance was evaluated in order to avoid the possibility of error and of reporting anomalies during calculation of direct costs. Baseline distribution of all variables included were evaluated in order to describe epidemiological situation during study period. All epidemiological analyses were performed using STATA software v.13; all test were two-side and p-value of 0.05 is considered statistically significant. We calculated the total number of records with echinococcosis related DRG code, subdivided by citizenship code. The CE patient was characterized using the individual anonymous code together with ICD-9 CE code at least once in the period of the study. For epidemiological purpose we defined as CE case the hospitalization (with CE in primary or secondary diagnosis) of a patient. To compute the Ministry of Health overall remuneration for CE, we identified CE hospitalizations as all records with CE in primary diagnosis. Furthermore the case fatality rate was calculated, using the discharge to death code of patients combined with CE primary diagnosis code.

### Direct cost calculation

The HDR is an economic tool used to quantify and justify the costs incurred by public and private hospitals for hospitalizations and medical and surgical treatments.

Discharge diagnosis for Echinococcosis is identified according to Major Disease Classification (MDC) as MDC 18: Parasitic and systemic infectious diseases, the diagnosis code 122.0–122.9 Echinococcosis. In particular: 122.0 “Echinococcus granulosus infection of liver”, 122.1 “Echinococcus granulosus infection of lung”, 122.2 “Echinococcus granulosus infection of thyroid”, 122.3 “Echinococcus granulosus infection, other”, 122.4 “Echinococcus granulosus infection, unspecified”, 122.5 “Echinococcus multilocularis infection of liver”, 122.6 “Echinococcus multilocularis infection, other”, 122.7 “Echinococcus multilocularis infection, unspecified”, 122.8 “Echinococcosis, unspecified, of liver”, 122.9 “Echinococcosis, other and unspecified”. In Italy, reimbursements are detailed in Department of Health legislation concerning the remunerations paid out to hospital care services for acute cases as well as for rehabilitation and long-term care for post-acute and specialist outpatient care. From January 1997 to December 2012 it was regulated by the rate table of the Ministry decree of June 30 1997 on annex 1, while since January 2013 it has been updated with the annex of the decree of December 18 2008 amended on October 18 2012. The codes for reimbursement from 1997 to 2013 were: MDC 423 DRG 18 Type M (medical) “Diagnosis related to infectious and parasitological diseases”, MDC 415 DRG 18 Type C (surgical) “Surgery for infectious and parasitological diseases”; since 2013 MDC 423 DRG 18 Type M (medical) “Diagnosis related to infectious and parasitological diseases” and MDC 578 DRG 18 Type C (surgical) “Surgery for infectious and parasitological diseases” [27].

The amount of government remuneration at the time of discharge is linked with primary diagnosis; therefore, only patients with primary diagnosis codes ICD9-CM 122.0–122.9 were considered for this computation. Payment distribution is further divided into: treatment type (surgical intervention or medical treatment), ordinary hospitalization (OH) (over 1 day and up to 80 days), ordinary hospitalization lasting one day or less due to the patient being transferred or deceased, and day hospital (DH).[28]. Since individual HDRs were available, to compute direct costs there was no need to group patients by age or by year of hospitalization. The total costs was subdivided by NUTS2 and by citizenship. Direct costs and number of hospitalizations over the 2001–2014 period were graphically represented, the Average Annual Cost (AAc) per 10^5^ inhabitant, based on mean population in the study period was calculated [29].

### Disability-Adjusted Life Years (DALYs) calculation

All Italian patients with CE diagnosis code were considered for DALYs calculation.

Using HDRs individual code we were able to extract each single patient’s data through case history and to calculate the duration of the disease for each patient. The definition of Disability-Adjusted Life Years is:

DALY=YLL+YLD

In this study, as in recent GBD studies the age weighting and discount were not used, in this way:

YLL=yearsoflifelostcalculatedasSEYLL(standardexpectedyearsoflifelost)

for each fatal case.

YLD=DisabilityWeight(DW)*durationofillnessforeachpatient.

The value of DW = 0.123 for CE is chosen in accordance to Torgerson et al. 2015 and WHO methods and data sources for the global burden of disease estimates 2000–2011. [17] [30]. The computation of total DALYs for a given sequela of a given illness is achieved by sum of individual DALYs.

## Results

**Fig 1:**
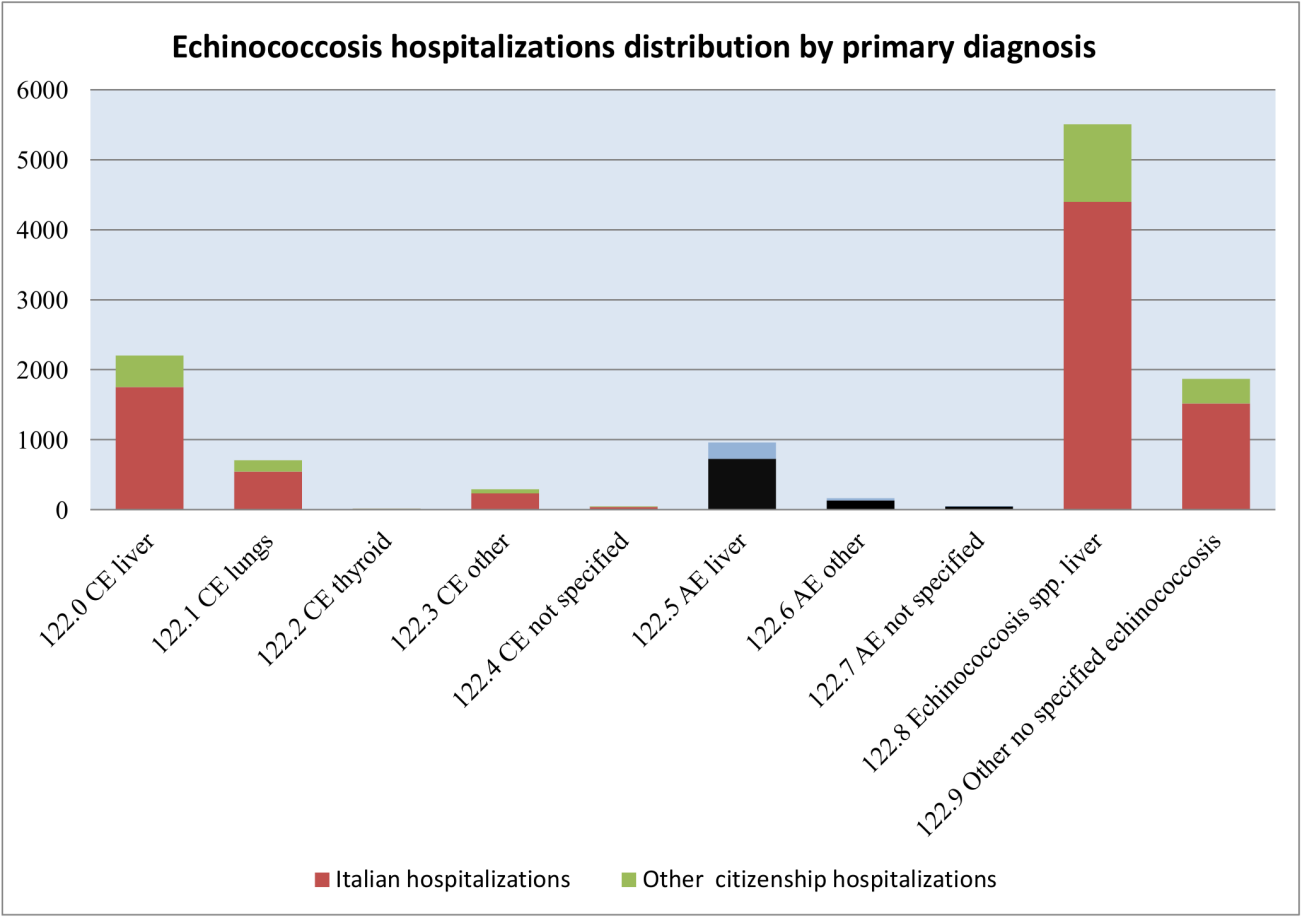
Echinococcosis hospitalizations distribution by primary diagnosis.

**Fig 2:**
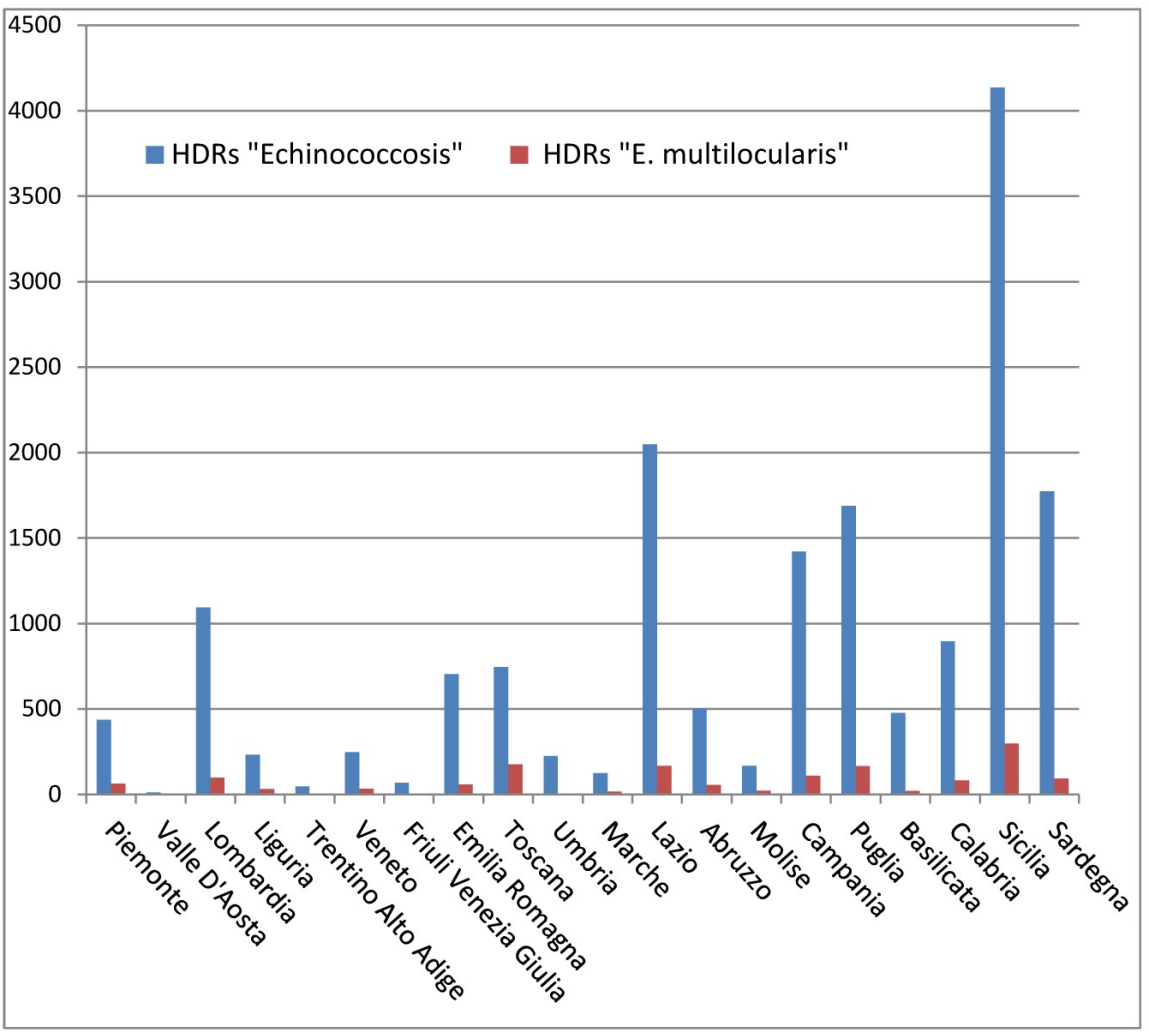
Echinococcosis HDRs distribution by region.

This study delineates the economic picture of the costs of public health and the burden of this disease, calculated on the basis of HDRs, for the Human Cystic Echinococcosis in Italy from 2001 to 2014. First we analyzed the epidemiological situation, then we proceeded to calculate direct cost of hospitalizations and to the DALYs calculation. An analysis of 21,050 HDRs evidenced the presence of 12,619 patient (10,901 Italian patients and 1,718 foreign patients).The surgical patients amount to 3,634 and the medical patients were 8,967. The case fatality rate was 0,36% (SE 0.05%). The HDRs of patients with Italian citizenship and with discharge diagnosis code referred to Echinococcus multilocularis (ICD-9 code 122.5–122.7) are 903 related to 784 patients (Fig 1). These records can be recognized as misclassifications, with a diffuse distribution throughout the Italian territory and with the average regional frequency of 9.96% (±2.92 C.I. 95%) (Fig 2). These diagnoses can be defined errors because both official data and recent and past literature fail to demonstrate the presence of human AE in Italy [4].

### Epidemiological situation

Tables 1 and 2 show epidemiological situation about CE disease in Italy, from 2001 to 2014, divided by Italian and foreign patients, while the economic evaluation is differently performed. Prevalence per 100000 inhab/year has been calculated and constant decrease of Annual Average of prevalence from 2.78 cases/100.000 inhabitants/year in the 2001 (CI 95% 2.64–2.92) to 1.06 in the last year (CI 95% 0.98–1.16) was observed. Number of cases and all patients’ characteristics are presented by sex, in order to evaluate possible different disease distribution. Starting from 21,050 records 12,619 cases were defined as indicated in Ministry guidelines performed in 1992 (Ministerial Decree July 26th, 1993) and update in 2008 (Ministerial Decree October 23th, 2008). Focusing on Italian patients, a greater number of cases are reordered in male patients over 50 years, especially in the South and Islands. Patients affected by CE are mostly treated with medical therapy (72%) and the length of stay is about 8 days (median = 8; I-III quartile = 4–13). The type of discharge is contain in the HDRs (death, ordinary discharge, discharge at nursing home (RSA), discharge to home with activation of home care, voluntary resignation, and transfer). A total of 162 patients with CE were discharged to death during 2001–2014, but it’s important to emphasize that all of them were suffering from other diseases, of which in 46 the primary diagnosis was CE: 20 fatal cases with DRG code 122.8, 7 with DRG code 122.0, 6 with DRG code 122.5, 6 with DRG code 122.9, 5 with DRG code 122.1, 1 with DRG code 122.3, 1 with DRG code 122.7. The average age at death was 63.61 (95% CI = 57.8–69.4).

**Table 1:** Baseline characteristics of Italian HDRs, by sex.

	Male*	Female*	Overall*
**Records**	9559 (54%)	8240 (46%)	17799
**EC patients**	5635 (52%)	5205 (48%)	10840
**Age (years)**			
<15	92 (1.7%)	70 (1.4%)	132 (1.5%)
15–20	81 (1.4%)	65 (1.2%)	146 (1.3%)
20–25	82 (1.4%)	91 (1.7%)	173 (1.6%)
25–30	129 (2.3%)	148 (2.8%)	277 (2.6%)
30–35	215 (3.8%)	189 (3.6%)	404 (3.7%)
35–40	256 (4.5%)	233 (4.5%)	489 (4.5%)
40–45	345 (6.1%)	254 (4.9%)	599 (5.5%)
45–50	321 (5.7%)	284 (5.5%)	605 (5.6%)
50–55	412 (7.3%)	399 (7.7%)	811 (7.5%)
55–60	455 (8.1%)	423 (8.1%)	878 (8.1%)
60–65	550 (9.8%)	497 (9.6%)	1047 (9.7%)
65–70	726 (12.9%)	547 (10.5%)	1273 (11.7%)
>70	1971 (35.0%)	2005 (38.5%)	3976 (36.7%)
**NUTS 1**			
North-west	633 (11.2%)	550 (10.6%)	1183 (10.9%)
North-east	351 (6.2%)	341 (6.6%)	692 (6.4%)
Center	1007 (17.9%)	975 (18.7%)	1982 (18.3%)
South	1736 (30.8%)	1619 (31.1%)	3355 (30.9%)
Islands	1908 (33.9%)	1720 (33.1%)	3628 (33.5%)
**Type of treatment**			
Medical	4062 (72.2%)	3773 (72.6%)	7835 (72.4%)
Surgical	1566 (27.8%)	1421 (27.4%)	2987 (27.6%)
**Length of stay**°	8 [4–14]	8 [4–13]	8 [4–13]

*data are presented as number (percentage) or median [I-III quartile]

°length of stay is define as number of hospitalization days, if inpatient is equal to recovery, or number of accesses if day-hospital.

**Table 2:** Baseline characteristics of Foreign HDRs, by sex.

	Male*	Female*	Overall*
**Records**	1815 (56%)	1435 (44%)	3250
**EC patients**	943 (53%)	836 (47%)	1179
**Age (years)**			
<15	58 (6.1%)	41 (4.9%)	99 (5.6%)
15–20	51 (5.4%)	40 (4.8%)	91 (5.1%)
20–25	105 (11.1%)	114 (13.6%)	219 (12.3%)
25–30	172 (18.2%)	99 (11.8%)	271 (15.2%)
30–35	143 (15.2%)	112 (13.4%)	255 (14.3%)
35–40	117 (12.4%)	87 (10.4%)	204 (11.5%)
40–45	112 (11.9%)	89 (10.6%)	201 (11.3%)
45–50	71 (7.5%)	73 (8.7%)	144 (8.1%)
50–55	51 (5.4%)	66 (7.9%)	117 (6.6%)
55–60	16 (1.7%)	36 (4.3%)	52 (2.9%)
60–65	20 (2.1%)	26 (3.1%)	46 (2.6%)
65–70	10 (1.1%)	22 (2.6%)	32 (1.8%)
>70	17 (1.8%)	31 (3.7%)	48 (2.7%)
**Nationality**			
Morocco	331 (35.1%)	205 (24.5%)	536 (30.1%)
Romania	170 (18.0%)	205 (24.5%)	375 (21.1%)
Tunisia	107 (11.3%)	46 (5.5%)	153 (8.6%)
Albania	72 (7.6%)	64 (7.7%)	136 (7.6%)
Rep. Moldova	23 (2.4%)	48 (5.7%)	71 (4%)
Macedonia	28 (3.0%)	21 (2.5%)	49 (2.7%)
Peru	11 (1.2%)	41 (4.9%)	52 (2.9%)
Serbia	27 (2.9%)	22 (2.6%)	49 (2.7%)
Other	174 (18.4%)	184 (22%)	358 (20.1%)
**NUTS 1**			
North-west	315 (33.4%)	235 (28.1%)	550 (30.9%)
North-east	257 (27.2%)	230 (27.5%)	487 (27.4%)
Center	225 (23.9%)	227 (27.2%)	452 (25.4%)
South	101 (10.7%)	103 (12.3%)	204 (11.5%)
Islands	45 (4.8%)	41 (4.9%)	86 (4.8%)
**Type of treatment**			
Medical	614 (65.1%)	518 (61.9%)	1132 (63.6%)
Surgical	329 (34.9%)	318 (38.1%)	764 (36.4%)
**Length of stay**°	9 [5–16]	8 [4–14]	9 [4–15]

*data are presented as number (percentage) or median [I-III quartile]

°length of stay is define as number of hospitalization days, if inpatient is equal to recovery, or number of accesses if day-hospital.

Data referred to foreign patients present some different distribution (i.e. most cases are registered in younger patients residents in the North), although those differences are probably related to immigration distribution (Report ISTAT, 2014). Foreign patients came from about 80 different countries, the frequency distribution by nationality evidenced citizen from: Morocco (536), Romania (375), Tunisia (153), Albania (136), Moldova Republic (71), Macedonia (49), Peru (52), Serbia Montenegro (49), other countries (358).

### Direct cost of hospitalization

**Fig 3:**
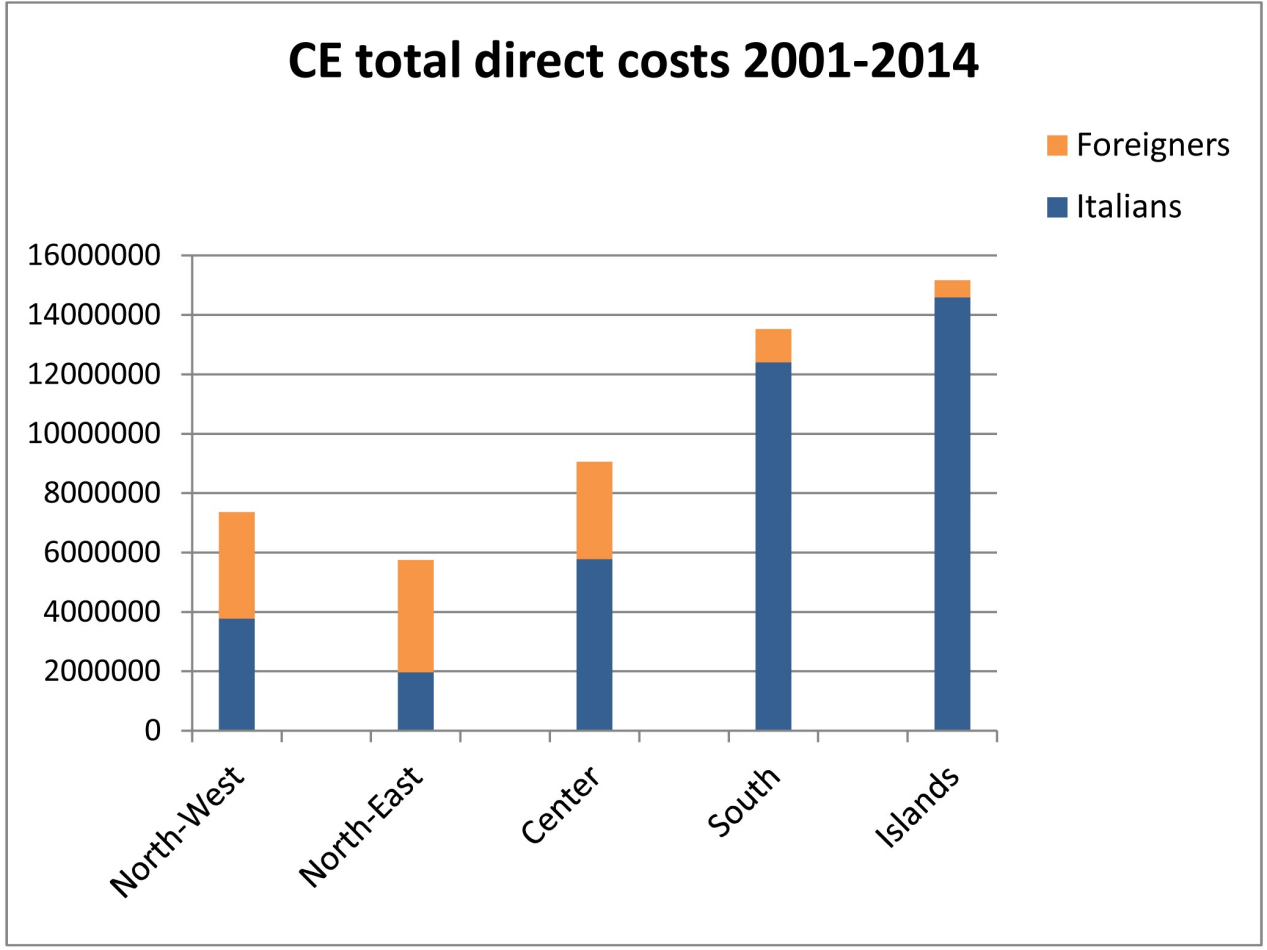
Direct costs of CE patients grouped by NUTS 1 for Italians and foreign patients in the study period.

The direct CE costs correspond to the overall remuneration provided by the national health services to hospitals. Human CE- associated economic losses that arise from diagnostic procedures, surgery and drug treatment, hospitalizations, periods of recovery, and fatalities are relatively easy to extrapolate from HDRs and enable the calculation of the direct costs of patients suffering from clinical echinococcosis. We considered 13,963 HDRs with primary CE diagnosis code of which 11486 HDRs related to Italian patients and 2477 to foreign patients. From 2001 to 2014, the National Health Service spent € 53175934,34 of which € 40828962,73 (76.78%) for Italian patients and € 12346971,612 (23.22%) for foreign patients, resulting in an average of around 3.8 million euros per year (SEM €250.000) (Table 2). The study of HDRs aimed at the assessment of economic remuneration by Regions grouped by NUTS 1 showed: the North-West spent € 7,366,074.68 of which € 3,772,170.36 (51.21%) for Italian patients and € 3,593,904.31 (48.79%) for foreign patients; the North-East spent € 5,748,540.81 of which € 1,969,050.96 (34.25%) for Italian patients and € 3,779,489.86 (65.75%) for foreign patients; the Center spent € 9,056,008.55 of which €5,775,066.89 (63.77%) for Italian patients and € 3,280,941.66 (36.23%) for foreign patients; the South spent € 13,523,999.37, of which 12,409,055.2 (91.76%) for Italian patients and € 1,114,944.17 (8.24%) for foreign patients; the Islands spent € 15,171,738.67, of which € 14,594,047.07 (96.19%) for Italian patients and € 577,691.60 (3.81%) for foreign patients. An amount of € 2,309,572.26 were related to HDRs with incorrect residence regional code. The details of total costs subdivided by NUTS 1 are illustrated in Fig 3.

**Fig 4:**
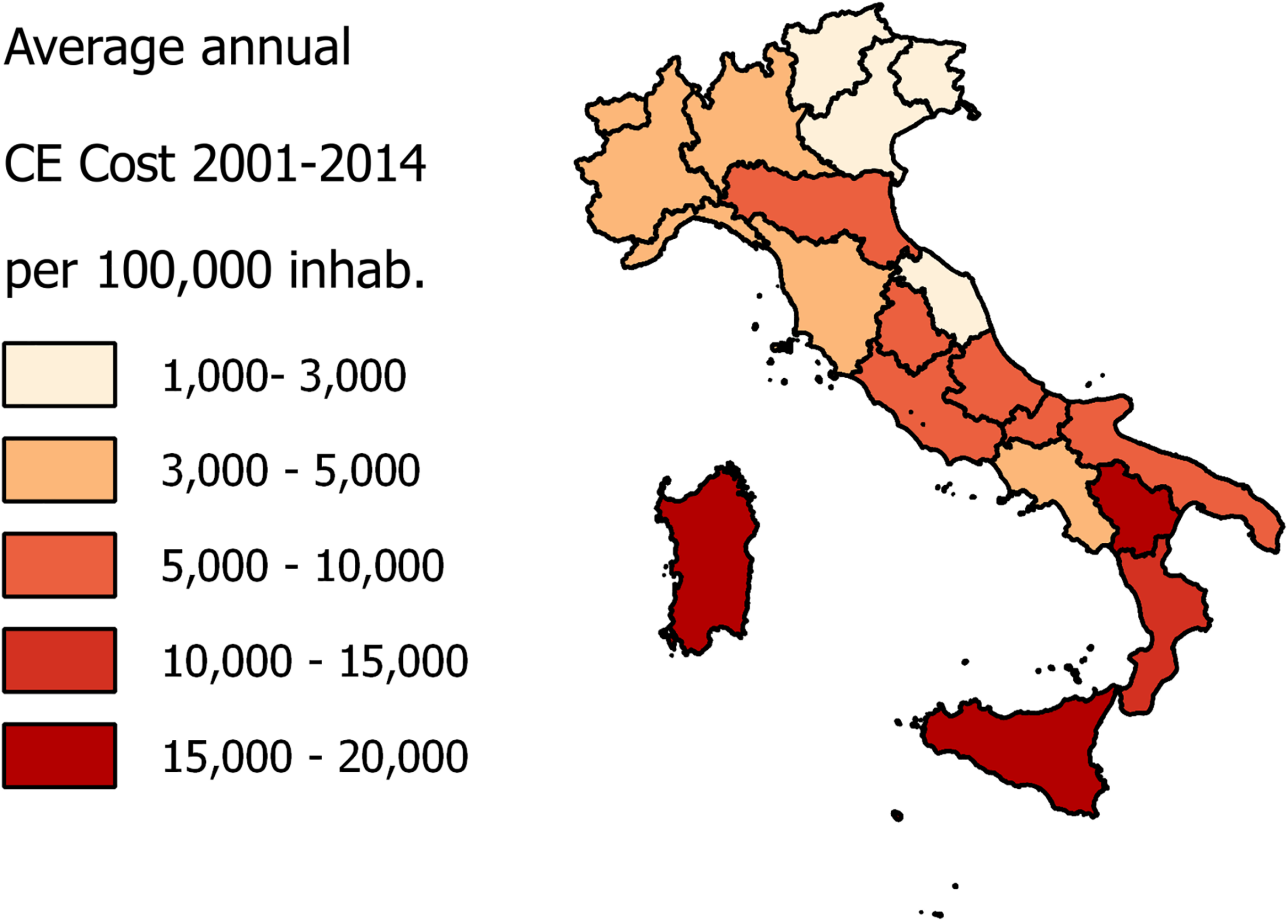
Choropleth map of average annual CE direct costs /100,000 inhabitants (2001–2014) by NUTS 2.

Fig 4 represents the average annual CE direct costs/100,000 inhabitants analyzed by NUTS 2. The average treatment cost for patients with primary diagnosis of CE amounted to 3734.43 € for Italians and 7186.82 € for foreigners. The average cost was € 8,152 per surgical case and € 2,458 per medical case treatment. The average Italian annual economic burden of CE in 2001–2014 was € 6,398 (SEM 421,18) per 100,000 inhabitants.

### DALYs

**Fig 5:**
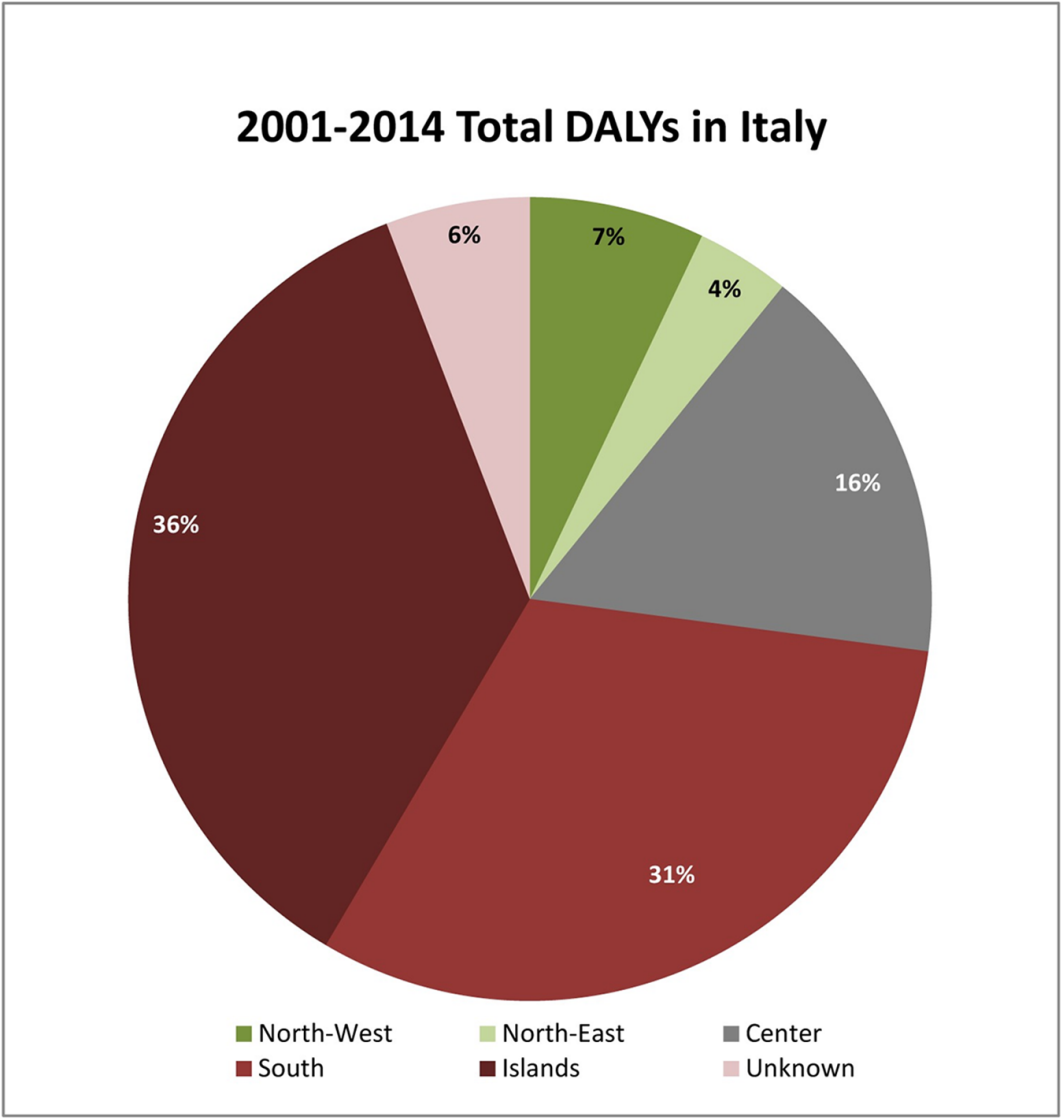
Graphic display of percentage Dalys by Italian NUTS 1.

Total DALYs for the 10,901 Italian patients considered over the 14 year study period were 3127.71 (223.4 DALYs per year) with 0.287 average DALYs per patient. DALYs subdivided by NUTS 1 are 220.43 (10.79%) in the North West, 118.36 (27.1%) in the North East, 508.42 (16.22%) in the Center, 982.13 (31.40%) in the South and 1,116.63 (35.95%) in the Islands, as shown in Fig 5. The geographical distribution for 181.73 DALYs (5.63%) was impossible to assess because of the incorrect residence regional code in some HDRs.

The years of life lost (YLL) in the study period are 1003.62 (32.09%), with an average of 25.73 (SEM 2.69) YLL for fatal case; the total number of years lived with disability (YLD) are 2124.09 (67.91%), with an average of 0.195 (SEM 0.002) YLD per patient.

**Table** 3. Direct costs and Dalys grouped into Nomenclature of Territorial Units for Statistics.

**Table 3:** Direct costs and Dalys grouped into NUTS 1 and NUTS 2.

	NUTS2	Regions	Inhab.Average 2001–2014	Direct Cost€	Totaldirect cost €	AA CEdirect cost€/10^5^ inhab.	Dalys		Total dalys annually /10^5^ inhab.		Total dalys annually	
**NUTS1NW**	**1**	Piemonte	4364947	**2120874.65**	7366074.68	3470,627	70.756	220.427	0.116	0.100	5.054	15.745
	**2**	Valle D'Aosta	125465	**66944.64**		3811,23	1.107		0.063		0.079	
	**3**	Lombardia	9627432	**4247281.92**		3151,176	124.21		0.092		8.872	
	**7**	Liguria	1591092	**930973.47**		4179,4	24.354		0.109		1.740	
**NUTS 1NE**	**4**	Trentino Alto Adige	1009052	**269441.018**	5748540.81	1907,314	7.872	118.361	0.056	0.074	0.562	8.453
	**5**	Veneto	4802338	**1856251.224**		2760,934	32.317		0.048		2.308	
	**6**	Friuli Venezia Giulia	1215947	**331560.98**		1947,694	5.535		0.033		0.395	
	**8**	Emilia Romagna	4287045	**3291287.594**		5483,777	72.637		0.121		5.188	
**NUTS1CENTER**	**9**	Toscana	3660583	**2408841.29**	9056008.55	4700,347	68.388	508.42	0.133	0.313	4.885	36.316
	**10**	Umbria	876420	**718209.22**		5853,433	24.108		0.196		1.722	
	**11**	Marche	1525502	**427075.888**		1999,697	15.621		0.073		1.116	
	**12**	Lazio	5522867	**5501882.156**		7115,717	400.303		0.517		28.593	
**NUTS1SOUTH**	**13**	Abruzzo	1312454	**1568346.56**	13523999.36	8535,519	114.483	982.13	0.623	0.498	8.177	70.152
	**14**	Molise	318292	**441580.108**		9909,591	24.477		0.549		1.748	
	**15**	Campania	5797102	**3490158.712**		4300,374	236.077		0.291		16.863	
	**16**	Puglia	4065597	**3768924.134**		6621,632	301.362		0.529		21.526	
	**17**	Basilicata	587581	**1336387.884**		16245,64	89.235		1.084		6.374	
	**18**	Calabria	1994771	**2918601.97**		10450,9	216.496		0.775		15.464	
**NUTS1ISLANDS**	**19**	Sicilia	5027388	**10648137.86**	15171738.66	15128,76	638.45	1116.634	0.907	1.194	45.604	79.760
	**20**	Sardegna	1654979	**4523600.81**		19523,77	478.184		2.064		34.156	
**UNKNOWN**				2309572.26			181.735				12.981	
**National TOTAL**			59366855	**€ 53175934.34**			**3127.707**		**0.376**		**223.408**	

## Discussion

Nowadays in Italy no CE surveillance system is in place. The Italian registry of CE (RIEC) was launched only in 2012. RIEC is a prospective multicentre registry of CE patients that have been visited since January 2012 in Italian health centers; data are voluntarily submitted to the registry [31]. RIEC was integrated in the European Register of Cystic Echinococcosis (ERCE), launched in October 2014 in the context of HERACLES project of the European Union Seventh Framework Programme (FP7/2007-2013) [32]. ERCE is a European prospective, observational, multicentre registry of patients with suspected or confirmed CE diagnosis. In Italy no CE screening studies were performed in the last years, either in adults or in schoolchildren [33]. Further national studies are needed in order to evaluate the prevalence of undiagnosed CE. Two out of five WHO collaboration centers for echinococcosis are in Italy, one at the University of Pavia, for Clinical Management of Cystic Echinococcosis, the newest one at Istituto Superiore di Sanità in Roma, finalized to the study of epidemiology, detection and control of cystic and alveolar echinococcosis (in humans and animals) [19]. Unfortunately, in the Italian context, the use of the adjective "neglected disease" for CE is particularly fitting for the current unavailability of extracting information from official database [20]. In the previous study conducted by National Reference Laboratory for CE (CeNRE) it was clear that HDR-based studies are a useful tool to evaluate the disease epidemiology, nevertheless it cannot replace a surveillance system implemented by official notifications [21]. Previous studies assessed the accuracy and the HDR’s contribution in estimating prevalence of different diseases, comparing this data source with others (i.e. drug prescriptions, health-tax exemption, causes of death, cancer registries); depending on the disease, those studies demonstrated that the absolute contribution given by HDRs is between 8–72% of all disease cases [34] [35] [36]. In their paper, Rosso and Zanetti [37] underline the great diffusion of HDRs studies and the importance of HDRs information by pathology to use it as database; they also consider the HDRs as an appropriate tool for economical and programming studies. Since cooperation for the implementation of RIEC in 2012, the CeNRE intention has always been to support the effort of the two WHO collaborating centers in Italy in order to have an Italian epidemiological picture as accurate as possible of spread and disease distribution in animals and humans. However, in the use of HDRs there are some limiting factors, such as the ability of the physician to provide a correct diagnosis, and the unknown proportion of cases managed as outpatients by “watch and wait” approach according to the “Expert Consensus for the diagnosis and treatment of cystic and alveolar echinococcosis in humans” and hence unreported. Indeed, our study aims to evaluate the economic impact on the finances of Italian Public Health Service through the calculation of the total remuneration for the ICD-9 codes related to echinococcosis in the period 2001–2014 and providing first set of processed data, useful as starting point for comparison with data acquired in the future [38] [39] [21]. Finally, remarking again the underreporting and the high number of mistakes in the management of records, we aim to suggest, for the first time in Italy with the support of national economical data, the need for an improved ad hoc information flow for this neglected disease.

### The data

The HDRs database is the only official database that covers all fourteen years, from 2001 to 2014, making it suitable for long-term studies and maximizing the chance of inclusion of undiagnosed or asymptomatic cases [34]. Data were extracted from all HDRs with at least one Cystic Echinococcosis diagnosis, either in the primary diagnosis or in one of the five possible secondary diagnoses. This allow for including many cases diagnosed by chance and many patient with developed disease prior to the study period. We did not consider asymptomatic patients since it is not possible assess their reimbursement costs, also they do not fit in to computation of DALYs because they do not qualify as “people living with disability”. The estimation of HDRs underreporting in Italy will be better achieved after full development of National and European Registry of Echinococcosis that could allow for linkage studies aimed to quantifying accuracy of databases [40].

### Direct costs calculation

**Fig 6:**
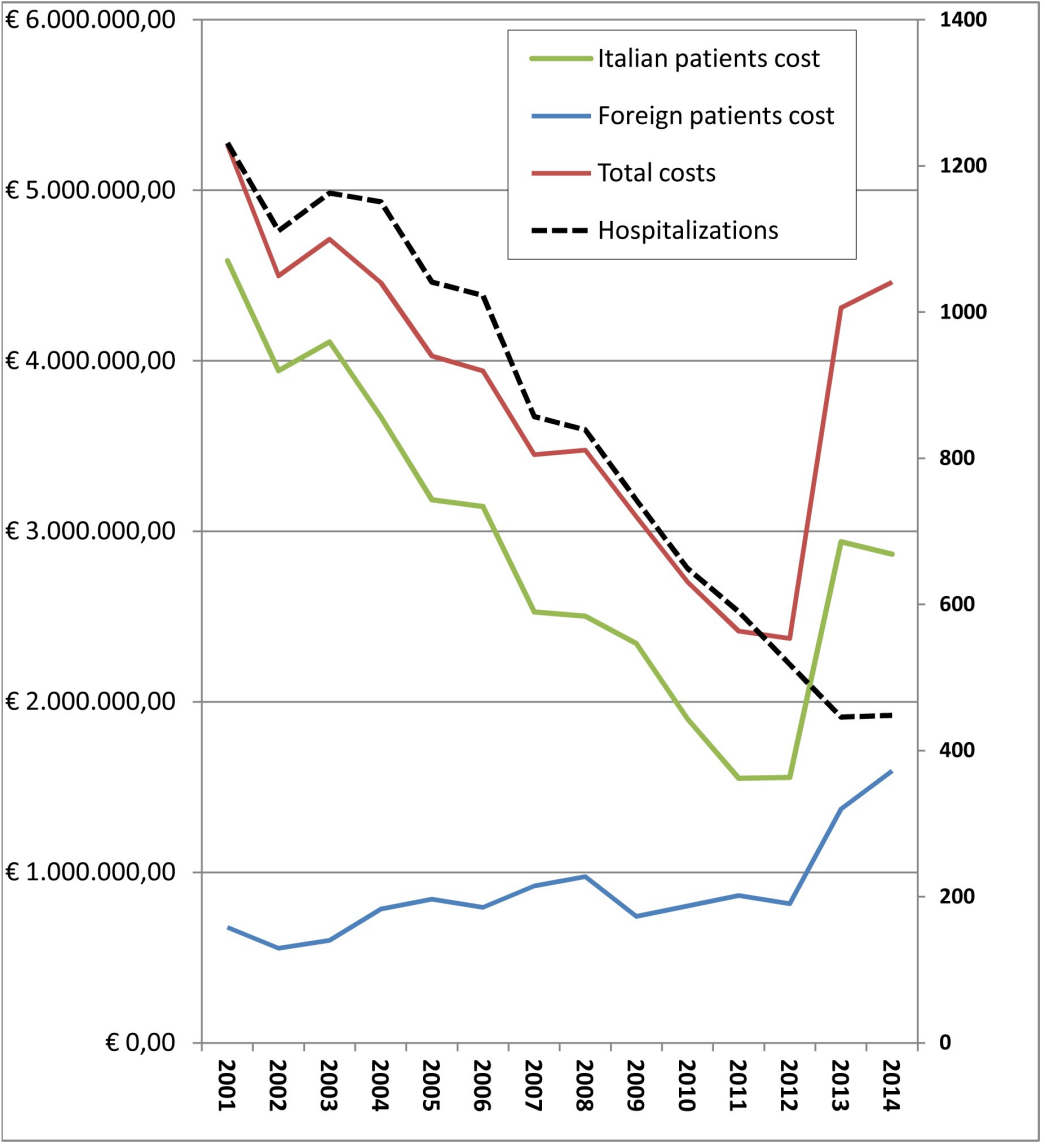
Representation of direct costs (left vertical axis) and number of hospitalizations (right vertical axis) over the 2001–2014 period.

The considerations about burden of CE must not exclude an assessment of the economic impact of this disease upon the National Health System. The economic evaluation of the costs incurred in Italy for this disease was based on institutional economic repayment of the National Public Health System; the HDRs were provided by the Italian Ministry of Health. The total costs in the 14 years of the study amounted to around 53 million euro. The trend in costs was influenced by both the number of hospitalizations with CE primary diagnosis and the remuneration value fixed by the Health ministry. From January 1997 to December 2012 the reimbursements were regulated by the rate table ex DM 30/06/1997 annex 1, since January 2013 it has been updated by the annex of the law of December 18, 2008 amended on October 18, 2012. Updates of prices table increased remunerations up to 162% (e.g. surgical intervention from € 6,972.68 to € 18,314.00). In fact, as the graph suggests, costs followed a negative trend from 2001 to 2012 and a peak in 2013 (Fig 6). In their paper, Narra et al. found out that for 15 surgical patients treated in single center from 2008 to 2014, the surgical intervention related costs ranged from 5,874 to 23,077 per patient, the present study analysed 4239 surgical hospitalization of national database for 14 years, giving for a CE related surgical procedure an average cost of € 8,152; considering the update of remuneration prices across the study period, these findings are not in contradiction [41].This study show that the geographical breakdown of costs were strictly related to geographical distribution of disease conducted in a previous study with peaks in NUTS 1 Islands and Southern Italy and less financial commitment in the North [21]. There was an even more marked difference in CE costs related to the population in the various Italian regions (Fig 3, Fig 4). In NUTS 1 the ratio of Italian and foreign patients is different, with a substantial balance of hospitalizations among Italian and foreign patients in northern Italy and on the other hand with a high prevalence of hospitalizations of Italian patients in the South and in the Islands. This reflects the distribution of foreigners in the Italian territory, with a greater presence in the North and Central NUTS 1 against a minor presence in the South and Islands (35.2% in the North-West, 26.6% in the Northeast, 24.2% in the Center, in the South and Islands 14%) [42] [43].

### DALYs

**Fig 7:**
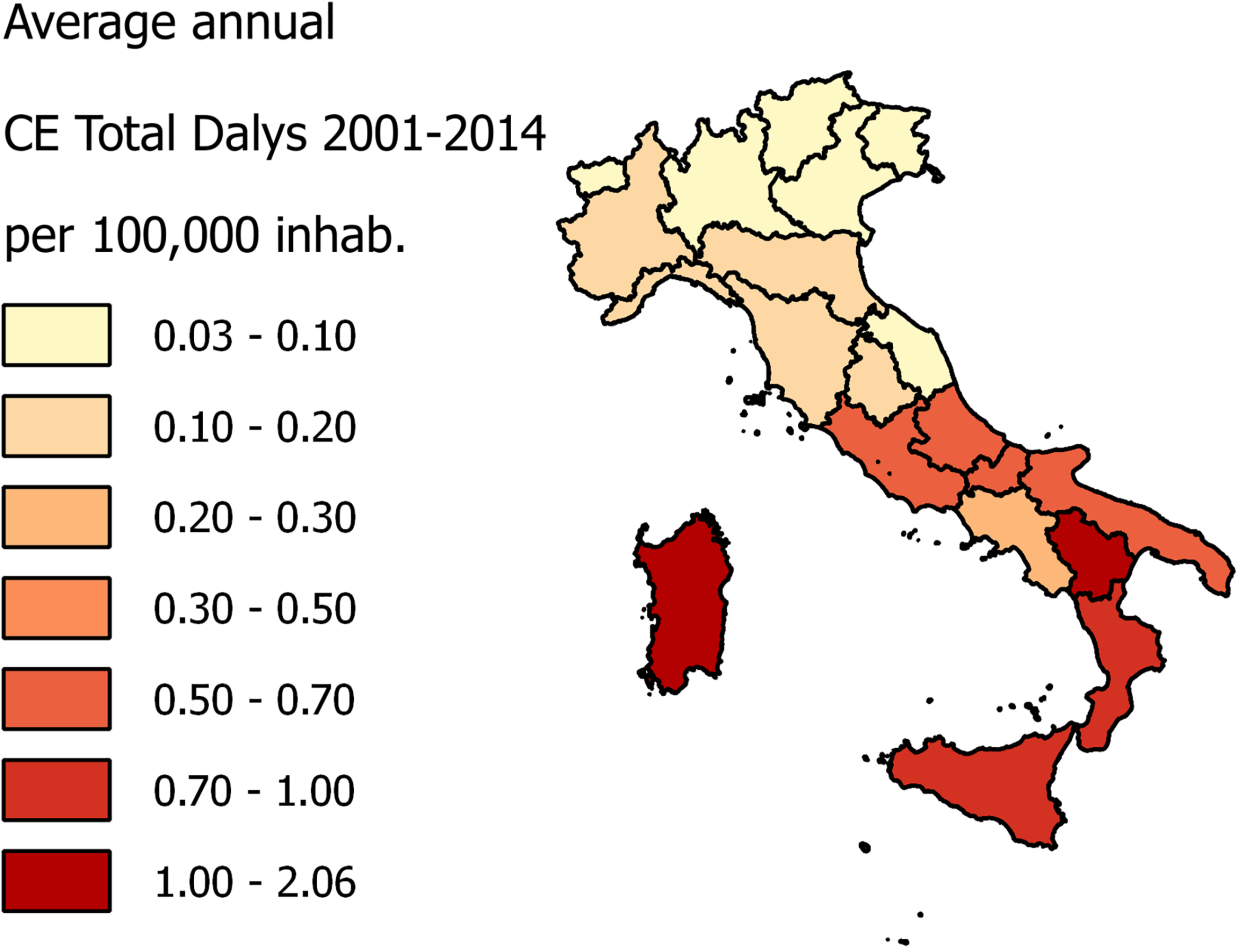
Choropleth map of average annual CE DALYs per 10^5^ inhabitants by NUTS 2.

The application of the DALYs formula (DALYs = YLL+YLD) revealed how in Italy this disease is in proportion rarely mortal (32% YLL), in fact this study showed a low case fatality rate (0.36%) and a high average age of fatal cases (63.61 years). The burden is mainly influenced by morbidity and duration (68% YLD). European DALYs median rate (95% uncertainty interval) per 100,000 inhabitants in 2010 reported by Torgerson et al. [44] was 0.8 (0.3–2); the Italian average annual DALYs in the study period per 100,000 inhabitants was 0.376 (SEM 0.035). Fig 7 represents geographical distribution by NUTS 2 of DALYs per 100,000 inhabitants in the study period. This confirm that Italy is part of the European CE endemic countries. Conducting an economic evaluation and a burden of disease study before the implementation of CE surveillance programs, should be the point of departure for a cost benefit study, that could stress the need of surveillance measures in high incidence areas [45].

## Supporting information

S1 Table HDR schema. (PDF)
