# Supplementary material for: The disease burden of human cystic echinococcosis based on HDRs from 2001 to 2014 in Italy
Source: PLoS Negl Trop Dis. 2017 Jul 26;11(7):e0005771. doi: 10.1371/journal.pntd.0005771 (PMC5546721; doi:10.1371/journal.pntd.0005771)
Supplement: S1 Table — (PDF) [file pntd.0005771.s001.pdf]

Field Name	Variable Name	Various
**cod_reg**	Hospitalization regional code	"010" = Piemonte; "020" = Val d'Aosta; "030" = Lombardia; "041" = P.A. di Bolzano; "042" = P.A. di Trento; "050" = Veneto; "060" = Friuli Venezia Giulia; "070" = Liguria; "080" = Emilia Romagna; "090" = Toscana; "100" = Umbria; "110" = Marche; "120" = Lazio; "130" = Abruzzo; "140" = Molise; "150" = Campania; "160" = Puglia; "170" = Basilicata; "180" = Calabria; "190" = Sicilia; "200" = Sardegna
**prov_res**	residence province code	ISTAT Codes
**com_res**	residence municipality code	ISTAT Codes
**sesso**	gender	"1" = Male; "2" = Female; "X" = Invalid data
**cittad**	cityzenship	"100" = italian citizenship; other citizenship by ISTAT codes
**età**	age	years
**reg_res**	residence regional code	"010" = Piemonte; "020" = Val d'Aosta; "030" = Lombardia; "041" = P.A. di Bolzano; "042" = P.A. di Trento; "050" = Veneto; "060" = Friuli Venezia Giulia; "070" = Liguria; "080" = Emilia Romagna; "090" = Toscana; "100" = Umbria; "110" = Marche; "120" = Lazio; "130" = Abruzzo; "140" = Molise; "150" = Campania; "160" = Puglia; "170" = Basilicata; "180" = Calabria; "190" = Sicilia; "200" = Sardegna
**usl_res**	regional hospital code	
**tip_att**	Activity Type	"A" = Acute; "N" = Nursery; "L" = Long-term care; "R" = Rehabilitation; "X" = errato
**reg_ric**	Hospitalization Type	"1" = Ordinary Hospitalization; "2" = Day Hospital; "X" = errato
**data_ric**	hospitalization date	yyyymmdd
**gg_deg**	hospital stay/ day hospital admissions	Length of stay in ordinary regime if reg_ric = "1"; Number of accesses in Day Hospital if reg_ric = "2"
**Tipo_drg24**	DRG type	"C" = Surgical DRG ; "M" = Medical DRG ; " " = not classified DRG
**codice**	identification code	Anonymous code for tracking repeated admissions; Use only class codes "A", "B", "C";
**dpr**	Primary Diagnosis	ICD9CM diagnosis code
**dsec1**	Secondary diagnosis 1	ICD9CM diagnosis code
**dsec2**	Secondary diagnosis 2	ICD9CM diagnosis code
**dsec3**	Secondary diagnosis 3	ICD9CM diagnosis code
**dsec4**	Secondary diagnosis 4	ICD9CM diagnosis code
**dsec5**	Secondary diagnosis 5	ICD9CM diagnosis code
